# Statement of Retraction: The amelioration of alcohol-induced liver and intestinal barrier injury by *Lactobacillus rhamnosus* Gorbach-Goldin (LGG) is dependent on Interleukin 22 (IL-22) expression

**DOI:** 10.1080/21655979.2026.2730731

**Published:** 2026-10-01

We, the Editors and Publisher of the journal *Bioengineered*, have retracted the following article from publication.

Ge, Y., Sun, H., Xu, L., Zhang, W., Lv, J., & Chen, Y. (2022). The amelioration of alcohol-induced liver and intestinal barrier injury by *Lactobacillus rhamnosus* Gorbach-Goldin (LGG) is dependent on Interleukin 22 (IL-22) expression. *Bioengineered*, 13(5), 12650–12660. https://doi.org/10.1080/21655979.2022.2070998

Since publication, significant concerns have been raised by a third party about the integrity of the data and reported results in the article. Specifically, concerns were raised about alleged image duplication with similar images from unrelated articles:

- Figure 4b first panel (model+LGG) appears to be duplicated with a similar image in Figure 3B EN+BAI/x40 panel from Zhao et al, 2021 (https://doi.org/10.1177/17534259211057038)
- Figure 4c β-actin band appears to be duplicated with a similar image in Figure 5i GAPDH band from He et al, 2022 (https://doi.org/10.1515/med-2022-0529)

When approached for an explanation, the authors did not respond to our queries and so these serious concerns remain unaddressed.

As verifying the validity of published work is core to the integrity of the scholarly record, we are therefore retracting the article. The authors have been informed of this decision.

We have been informed in our decision-making by our policy on publishing ethics and integrity and COPE guidelines. 

The retracted article will remain online to maintain the scholarly record and will be digitally watermarked on each page as ‘Retracted’. 

